# How Protein Stability and New Functions Trade Off

**DOI:** 10.1371/journal.pcbi.1000002

**Published:** 2008-02-29

**Authors:** Nobuhiko Tokuriki, Francois Stricher, Luis Serrano, Dan S. Tawfik

**Affiliations:** 1 Department of Biological Chemistry, Weizmann Institute of Science, Rehovot, Israel; 2 EMBL-CRG Systems Biology Partnership Unit, CRG-Centro de Regulacion Genomica, Barcelona, Spain; University of California Los Angeles, United States of America

## Abstract

Numerous studies have noted that the evolution of new enzymatic specificities is accompanied by loss of the protein's thermodynamic stability (ΔΔ*G*), thus suggesting a tradeoff between the acquisition of new enzymatic functions and stability. However, since most mutations are destabilizing (ΔΔ*G*>0), one should ask how destabilizing mutations that confer new or altered enzymatic functions relative to all other mutations are. We applied ΔΔ*G* computations by FoldX to analyze the effects of 548 mutations that arose from the directed evolution of 22 different enzymes. The stability effects, location, and type of function-altering mutations were compared to ΔΔ*G* changes arising from all possible point mutations in the same enzymes. We found that mutations that modulate enzymatic functions are mostly destabilizing (average ΔΔ*G* = +0.9 kcal/mol), and are almost as destabilizing as the “average” mutation in these enzymes (+1.3 kcal/mol). Although their stability effects are not as dramatic as in key catalytic residues, mutations that modify the substrate binding pockets, and thus mediate new enzymatic specificities, place a larger stability burden than surface mutations that underline neutral, non-adaptive evolutionary changes. How are the destabilizing effects of functional mutations balanced to enable adaptation? Our analysis also indicated that many mutations that appear in directed evolution variants with no obvious role in the new function exert stabilizing effects that may compensate for the destabilizing effects of the crucial function-altering mutations. Thus, the evolution of new enzymatic activities, both in nature and in the laboratory, is dependent on the compensatory, stabilizing effect of apparently “silent” mutations in regions of the protein that are irrelevant to its function.

## Author Summary

To perform its function, a protein must fold into a complex, three-dimensional structure that is maintained by a network of interactions between its amino acid residues. Evolution of a new protein function will be driven by mutation of amino acids in key positions (*new-function mutations*). Such mutation can also hamper interactions that ensure the stability of a protein's fold—sometimes to a degree that renders the protein non-functional. Indeed, previous studies have noted that the evolution of new enzymatic functions is accompanied by significant losses in protein stability, suggesting a “tradeoff” between acquisition of new enzymatic functions and stability. But since most mutations are destabilizing, we sought to compare new-function mutations with other types of mutations. We performed a comprehensive analysis of the type, location, and stability effects of mutations that have conferred new enzymatic functions in laboratory evolution experiments. We found that stability changes (ΔΔ*G*) of new-function mutations are similar to those of all other mutations, but are weaker than those of mutations that characterize neutral evolutionary changes (mutations that accumulate with no change of structure and function). Our analysis also revealed the important role of neutral (i.e., “non-functional”) mutations in compensating for the destabilizing effects of the “new-function” mutations.

## Introduction

With the exception of unstructured protein domains, the integrity of a protein's structure and function is largely dependent on its thermodynamic stability. Evolutionary processes, be they neutral, or adaptive, involve the acquisition of mutations that may affect protein function and/or stability. For example, a mutation that endows a desirable new function, but severely undermines stability, will not become fixed. The relationship between mutational effects, function and stability is therefore crucial to our understanding not only of the evolutionary dynamics of proteins [1]–[6], but also in engineering, designing, and evolving, novel enzymes in the laboratory [7]–[12].

Stability-function tradeoffs became originally evident in enzymes, particularly in the structural tension created by the arrangement of catalytic residues in active sites. From the point of view of overall protein stability, active site organization is inherently unfavorable for a number of reasons. Functional residues, which are generally polar or charged, are embedded in hydrophobic clefts [13], sometimes with proximal like charges. Key catalytic residues often possess unfavorable backbone angles [14],[15]. Consequently, the substitution of an enzyme's key catalytic side chains (typically into alanine) can dramatically increase stability whilst obviously sacrificing activity [16]–[23].

Such observations (notwithstanding exceptions such as residues that contribute to both function and stability [24]–[26], and cases where enzyme stability can be increased without comprising function [10], [27]–[31]) led to the generally accepted principle of stability-function tradeoffs [16],[19] that was later extended to tradeoffs between *new functions* and stability [32]. However, as discussed below, we surmise that there exists a fundamental difference between mutations in key catalytic residues that relate to the well established stability-function tradeoff, and mutations that mediate the evolutionary divergence of new functions.

Enzymes evolve new functions via mutations that alter substrate specificity, typically by increasing the affinity and rates for weak promiscuous substrates. These changes involve mutational adjustments of the active site, its periphery, or even the “second” and “third shell” of residues that surround it, while maintaining the key catalytic residues intact. As shown below, in oppose to mutations in key catalytic residues that typically involve an exchange into alanine of a charged/polar residue within a hydrophobic surroundings, the type and location of new function mutations is far more diverse. As initially observed by Wang et al. [32],[33], most mutations that confer new functions have been proven to be destabilizing (for recent examples see [34]). However, the generality of stability-function tradeoffs with regard to new functions should be addressed in view of the fact that, regardless of their relevance to function, *most* mutations are destabilizing [30], [35]–[37]. Indeed, derivation of the ΔΔ*G* distributions of all possible mutations in a series of globular proteins using the experimentally validated FoldX algorithm [38],[39]) indicated that about 70% of mutations are destabilizing (ΔΔ*G*>0 kcal/mol), and >20% are significantly destabilizing (ΔΔ*G*≥2 kcal/mol) [40]. On the other hand, mutations that characterize neutral, non-adaptive changes (mutational drifts with no changes in protein function and structure) occur primarily on the surface, certainly at the first steps of sequence divergence [41], and this subgroup of mutations is much less destabilizing (average ΔΔ*G* = 0.6 kcal/mol [40]. Thus, better understanding of how the emergence of new functions trades-off with protein stability requires a comparison of mutations that confer new protein functions to all other possible mutations in a protein, as well as to mutations that characterize neutral, non-adaptive changes.

With this in mind, we investigated a large set of mutations that were found in enzymes that acquired new substrate specificities in directed evolution experiments and clinical isolates (548 mutations in 22 different enzymes). We applied FoldX to compute the ΔΔ*G* values of these mutations, and compared the type (hydrophibicity/polarity), location (solvent accessibility and secondary structure assignment), and ΔΔ*G* values of these mutations with all possible point mutations in the same proteins. While realizing that the FoldX values are a prediction of limited accuracy, they do enable the examination the distributions of ΔΔ*G* values for a large set of proteins and mutations, and on the whole, these predictions show reasonable correlation with experimental data [40]. Thus, whilst the values for individual mutations can considerably deviate from the experimental values, the trends we observed are likely to be relevant [42].

## Results

### Classification of Mutations

We systematically explored the directed evolution literature from 2003 to date for cases amenable to our analysis. The criteria included enzymes in which few, or more, mutations accumulated, and a new substrate specificity evolved in response, and that have a high resolution crystal structure (a list of the analyzed enzymes and mutations is available as Table S1). TEM-1 mutations observed in clinical isolates, and subsequently in laboratory evolution experiments, were also included in our analysis.

Variants isolated in directed evolution experiments and clinical isolates generally possess multiple mutations. Nevertheless, as with natural enzymes, only some mutations are directly related to the newly acquired function, while others are largely neutral. The mutations in the studied enzyme variants were therefore classified into two categories: (a) *new-function* mutations—i.e., mutations that confer the new function, and (b) *other* mutations—i.e., all mutations that accumulated in these variants alongside the adaptive mutations. We assigned mutations as *new-function* mutations by three criteria: (i) the mutation was the only mutation in the variant showing the new activity or selectivity; (ii) the mutation was identified by the authors as *contributing* to the new function; (iii) the mutation was conserved, or dominant, in all the variants isolated after several rounds of mutation and selection. *Other* mutations included nonessential mutations that were seen in only one of the isolated variants, or were shown to be irrelevant to the functional change. Using these criteria, we classified 246 mutations as “*new-function*” mutations, and 302 mutations as “*other*” mutations (Table S2).

### Type and Location of the Mutations

The location and type of a mutated residue affects the stability changes induced by mutations in this residue. In particular, the distribution of ΔΔ*G* values differs significantly for surface *vs.* core residues. Thus, as the solvent accessibility (ASA) of a residue decreases, the destabilizing ΔΔ*G* values of its mutation increase [40]. It was therefore necessary to account for the location of “*new-function*” and “other” mutations and thus ensure a balanced comparison with all other possible mutations in residues of equivalent type and location.

#### Type of mutations

**Figure 1:**
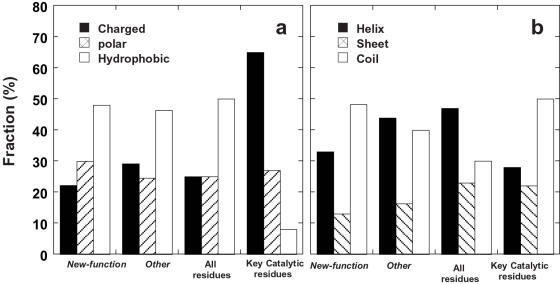
Distribution of mutations with respect to residue type (a), and their location in secondary structural elements (b). Noted are: *all* protein residues (referring to mutations attainable in all protein residues by single nucleotide exchanges); *key catalytic* residues (data adapted from Bartlett et al. [43]); and residues in which mutations identified in directed evolution experiments occur, divided to *new-function* mutations, and *other* mutations, as explained in the text.

The key catalytic residues of enzymes are generally charged or polar [43]. However, our analysis showed about 50% of *new-function* mutations involved changes in hydrophobic residues (Figure 1a). This proportion is very similar to that found for the *other* mutations, and indeed for *all* protein residues. The fraction of polar residues seems to slightly increased in *new-function* mutations, and a higher fraction of charged residues were exchanged by *other* mutations The latter correlates with the observation that other mutations tend to be in surface residues (see below). The fraction of hydrophobic residues seems to slightly increase after mutations in both the *new-function* (from 47% to 50%) and *other* mutations (from 47% to 48%) have been incorporated. This tendency might relate to biases in the mutagenesis methods employed [44]. Overall, this analysis indicated that, in contrast to key catalytic residues where charged and polar residues dominate, the types of residues in which *new-function* mutations occur are distributed in a manner similar to the rest of the protein.

#### Secondary structure

About 70% of the total residues in enzymes occur in secondary structures such as α-helices and β-sheets. The remaining 30% are found in random coils. In contrast, about 50% of active site residues are located in random coils [43]. In accordance, the *new-function* mutations are more often found in random coils than in α-helices and β-sheets, and those proportions are similar to key catalytic residues (Figure 1b). *Other mutations* are found less in random coils than *new-function mutations*, closer to *all* mutations (Figure 1b). This supports the fact that the *other mutations* are not directly involved in the acquisition of new function.

#### Solvent accessibility

**Figure 2:**
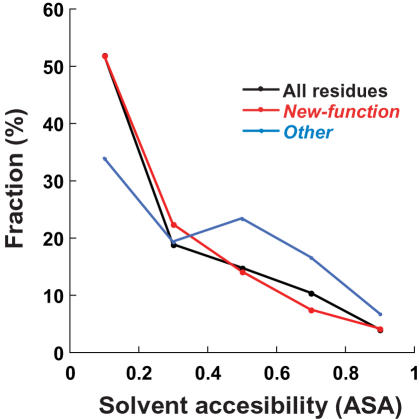
Solvent accessibility (ASA) of the mutated residues. ASA values were calculated with “ASA view” (http://www.netasa.org/asaview/). Plotted are the distributions for *all* protein residues, and residues in which *new-function* mutations, and *other* mutations occur.

In general, catalytic residues tend to be partially exposed to solvent [43]. However, the ASA values of *new-function* mutations are distributed in a manner similar to all residues, whereas *other* mutations tend to locate more to the enzyme surface than its core (Figure 2), thus indicating that *other* mutations are involved in a different process, most likely neutral, non-adaptive evolution. Contrary, *new-function* mutations are distributed similarly to all other mutations, and with significantly more mutations in the core (ASA<0.25) than observed with *other* mutations, thus implying their role in the acquisition of new functions, and their larger destabilizing effects.

Overall, therefore, the *new-function* mutations are in quite similar types and positions as all residues, with the exception that they have a greater tendency to be located on random coils. The *other mutations* show a strong tendency to be located on the surface.

### The ΔΔ*G* Distributions

The stability effects of mutations (ΔΔ*G*) were computed with the protein design software FoldX, whose force-field is based on empirical energy terms correlated with experimental ΔΔ*G* measurements [38],[39]. In a previous work, we found that ΔΔ*G* distributions of all possible mutations in globular, monomeric, single domain proteins of ≤340 amino acids can be described by a universal bi-Gaussian function with only one free parameter (the protein's chain length) [40]. The vast majority of enzymes analyzed in the study, especially those that possess large number of mutations, meet the above size criterion (Table 1). We have therefore compared the distribution of ΔΔ*G* values for *new-function* mutations, and *other* mutations, with the distributions for all possible mutations that are attainable by single nucleotide substitutions from the protein's wild type sequence (*all mutations*). Although certain variants carry multiple mutations, we based our tradeoff analysis on the ΔΔ*G* values of individual mutations. In nature, and frequently in the lab, function-altering mutations tend to accumulate one at a time, and are combined only in subsequent generations. Indeed, in most cases, ΔΔ*G* and functional effects of multiple mutations are largely additive [30],[45].

**Table 1:** Summary of average ΔΔ*G* and ASA values of mutations.

Name		All residues			*New-function* mutations			*Other* mutations		
Common name	Abbreviation	ΔΔ*G*	ASA	# of mutations analyzed	ΔΔ*G*	ASA	# of mutations analyzed	ΔΔ*G*	ASA	# of mutations analyzed
Aspartate aminotransfrase	AspATs	n.d	n.d		0.77±1.26	0.35±0.29	24	-0.08±1.46	0.31±0.30	85
*Burkholderia gladioli* esterase EstB	EstB	n.d	n.d		1.15±0.98	0.20±0.20	9	0.44±0.58	0.44±0.20	2
Cytocrome c peroxidase	CCP	n.d	n.d		0.91±1.69	0.42±0.40	6	0.36±1.71	0.44±0.28	25
Dienelactone hydrolase	DLH	n.d	n.d		0.33±0.87	0.19±0.22	5	0.70±1.35	0.34±0.13	6
*E. Coli* dihydrofolate reductase	DHFR	n.d	n.d		1.61±2.26	0.32±0.28	14	0.69±1.62	0.39±0.25	8
*E. coli* Glucosamine synthase	GlmS	n.d	n.d		1.32±2.20	0.26±0.43	3	0.56±0.80	0.53±0.32	7
*E. coli* KDPG aldolase	KDPG aldolase	n.d	n.d		0.83±1.62	0.34±0.25	9			
Extradiol dioxygenase	DoxG	n.d	n.d		0.85±2.10	0.06±0.07	11	0.06±1.55	0.39±0.31	10
Human carbonic anhydrase II	CAII	1.44±2.03	0.30±0.29	1539	0.50±0.82	0.11±0.12	2	0.12	0.35	1
Human Glutathione transderase	hGST	n.d	n.d		2.08±1.77	0.34±0.25	8	0.74±0.97	0.31±0.29	39
*Neisseria polysaccharea* amylosucrase	Amylosucrase	n.d	n.d		0.53±1.65	0.28±0.27	6	1.04±0.74	0.24±0.30	4
OmpT protease	OmpT protease	n.d	n.d		-0.79±6.32	0.06±0.07	2	-0.11±1.37	0.50±0.21	2
OPDA phosphotriesterase	OPDA	n.d	n.d		-0.75±2.40	0.30±0.18	10			
*P. aeruginosa* Lipase	Lipase	1.13±1.85	0.27±0.27	1694	0.83±1.36	0.36±0.30	46	0.14±1.27	0.33±0.24	9
*P. diminuta* phosphotriesterase	PTE	n.d.	n.d		-0.09±1.66	0.21±0.19	8	0.20±1.63	0.31±0.26	10
P450 2A6	P450 2A6	n.d	n.d		0.30±0.41	0.11±0.05	5			
PTE homology protein	PHP	n.d	n.d		2.57±3.37	0.22±0.21	3	0.43±0.63	0.34±0.48	2
Recombinant serum paraoxonase 1	rePON 1	1.39±1.76	0.28±0.28	1936	1.21±1.21	0.25±0.21	31	0.47±1.65	0.47±0.31	48
*S. clavuligerus* deacetoxycephalosporin C synthase	DAOCS	n.d	n.d		0.60±1.00	0.32±0.30	17	0.05±0.88	0.31±0.34	10
Tagatose-1,6-bisphosphate aldolase	TBA	n.d	n.d		1.01±1.04	0.13±0.12	4	1.48	0.10	1
TEM-1 β-lactamase	TEM-1	1.14±1.69	0.28±0.28	1546	1.27±1.59	0.24±0.26	18	0.20±1.80	0.30±0.28	30
Vanillyl-alcohol oxidase	VAO	n.d	n.d		-0.03±2.27	0.02±0.02	5	0.99±1.55	0.23±0.10	3
				**total**	**0.86±1.65**	**0.28±0.26**	**246**	**0.39±1.37**	**0.39±0.29**	**302**

Additional details regarding the analyzed proteins are provided in Supplementary Table 1.

n.d. – not determined.

**Figure 3:**
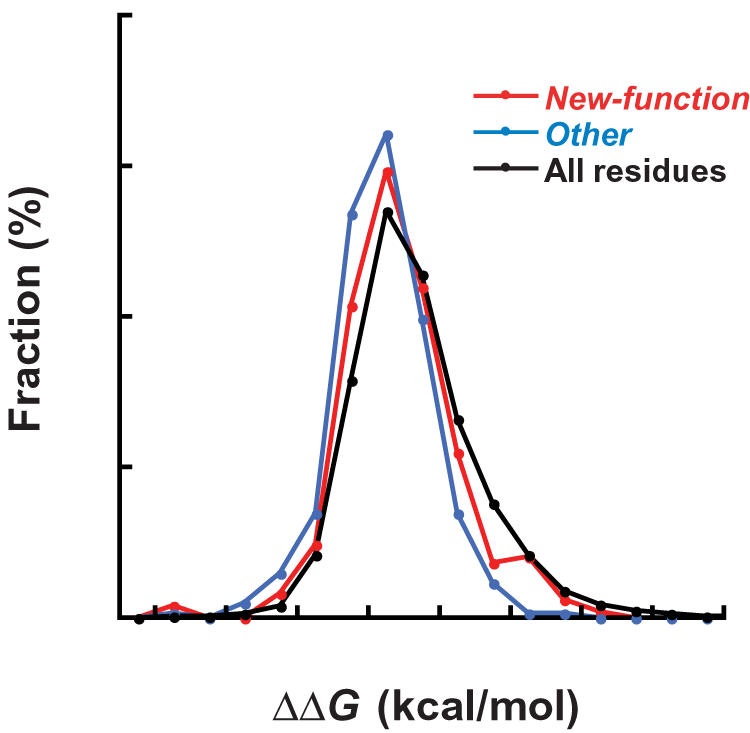
The ΔΔ*G* distributions of *new-function* mutations in comparison with *other* mutations and *all* possible mutations. The ΔΔ*G* values of mutations were computed by FoldX as described [38]–[40]. The resulting values were presented in histograms by classifying 25 bins, each 1.0 kcal/mol wide.

In all 22 enzymes analyzed here, the average ΔΔ*G* values for *new-function* mutations were found to be comparable to those of *all* mutations (Table 1). Overall, the distributions of ΔΔ*G* values for *new-function* mutations are nearly identical to those of *all mutations*, although there are significantly fewer highly destabilizing mutations (ΔΔ*G*>3 kcal/mol) in *new-function* mutations (8%) than in *all mutations* (15%) (Figure 3). This observation is expected as highly destabilizing mutations undermine the enzyme's structure and are therefore eliminated by selection, and is consistent with the analysis of ΔΔ*G* values of mutations that accumulated in a neutral drift under strong purifying selection (ΔΔ*G*≤3 kcal/mol) [46].

Because *new-function* mutations are distributed in different secondary structure elements than the rest of the protein (Figure 1b), their location might bias ΔΔ*G* distributions. To ameliorate this we adjusted the ΔΔ*G* distributions of *all* mutations to have the same proportion of secondary structure elements as *new-function* mutations by comparing the ΔΔ*G* distributions of random coils, α-helices, and β-sheets, of four of the studied enzymes (PON, CAII, Lipase, and TEM-1). The ΔΔ*G* values of β-sheets appeared to be more destabilizing than those of random coils and α-helices (Figure S1), as previously observed [47],[48]. Nevertheless, the overall effect of this adjustment was minor, and the distributions of ΔΔ*G* values for *new-function* mutations remained nearly identical to those of *all* mutations (Figure S1).

The overall picture that emerges is that the *new-function* mutations are distributed as all other mutations. The majority of both *all-* and *new-function* mutations are destabilizing (43% of mutations exhibit ΔΔ*G* values higher than 1 kcal/mol), and a significant fraction of mutations are actually stabilizing (7% of mutations exhibit ΔΔ*G* <−1 kcal/mol). Thus, the mutations associated with the acquisition of new functions are as destabilizing as the “average mutation”.

However, the *other* mutations (those that accumulated in variants alongside function altering mutations) are distributed in a different manner. They contain many more neutral, and stabilizing mutations, and fewer destabilizing mutations (30% >1 kcal/mol) than *all-* and *new-function* mutations (Figure 3). This distribution indicates that *other* mutations largely reflect neutral, non-adaptive evolution, whereby destabilizing mutations are purged out. Nevertheless, the significantly higher fraction of stabilizing mutations 38% <0 kcal/mol *vs.* 21% in *all* mutations) indicates that *other* mutations can also play a role in increasing protein stability, and thereby compensate for the destabilizing effects of the *new-function* mutations that drive the adaptive process.

## Discussion

### Do New Functions Tradeoff with Stability?

It is widely accepted that active site construction is thermodynamically unfavorable. Thus, many active site mutations, and the removal of key catalytic residues in particular, dramatically stabilize enzymes at the expense of activity [16]–[23]. By the same logic, stability is likely to be compromised when enzymes acquire new activities by evolutionary processes. To date, this hypothesis was supported by several sets of experimental data, but lacked a comprehensive analysis that compares the distribution of ΔΔ*G* effects of mutations that drive the acquisition of new functions over all other mutations. A comprehensive analysis of this kind can only be performed computationally simply because of the vast number of mutations that need to be analyzed. Although the computed FoldX values are of limited accuracy, they do enable the examination the distributions of ΔΔ*G* values for a large set of proteins and mutations, and on the whole, these predictions show reasonable correlation with experimental data [38],[40]. The computed average of ΔΔ*G* values for mutation endowing new functions (+0.9 kcal/mol) is also within the range of experimental values obtained for such mutations; the average of ΔΔ*G* value for six mutants that conferred new function in TEM-1 β-lactamase is +1.7 kcal/mol (+0.22 to +4.04 kcal/mol) [32].

The computational analysis indicated that *new-function* mutations are as destabilizing as mutations in other parts of the protein, and thus, there seems to be no distinct tradeoff between new functions and stability. Sanchez et al. have recently reached a similar conclusion by analyzing the correlation between ΔΔ*G* values and the frequency of mutations in functional sites of natural proteins. They found that selection for function is overruling selection for stability, but observed no anti-correlation between function and stability [49]. The above said, we also found that the type and location of new-function mutations are almost indistinguishable from the rest of the protein (other than a tendency to locate to random coils). That the solvent accessibility of *new-function* mutations is distributed as the rest of the protein (Figure 2) is indicative of their special nature. Neutral (non-adaptive) drift (i.e., the gradual accumulation of mutations while retaining function or structural) initially involves surface residues [41],[50], and thus minor stability changes. This is also reflected in the nature of the *other* mutations that tend to be on the surface and exhibit minor stability changes, and even stabilizing, compensatory effects as discussed below. In contrast, the acquisition of new-function involves also core residues, and is therefore more demanding in stability terms than a neutral drift. In that respect, i.e., when comparing neutral, to adaptive evolutionary changes, one could say that new function does trade-off with stability.

The tendency of *new-function* mutations to locate to random coils is also in accordance with the notion that the routes leading to new functions do not usually involve modification of either the enzyme's scaffold or key catalytic residues, but rather involve multiple, and often subtle, changes in loops that comprise the substrate binding pocket [51]–[53]. Indeed, directed evolution experiments indicated that most *new-function* mutations are located relatively far from the key catalytic residues, often being found in the periphery of the active site [51], [54]–[56]. Thus, the changes that drive divergence towards new functions do not usually involve the incorporation of the same type of thermodynamically unfavorable active site residues that provide the main catalytic function of the enzyme. Indeed, in enzyme superfamilies, despite a wealth of different reactions and substrates, scaffolds and key catalytic residues remain unchanged [57].

Our analysis therefore indicates that the two classes of residues—i.e., key catalytic residues, and new function residues, are subject to different rules. Key catalytic residues are inherently, and dramatically destabilizing, and therefore exhibit distinct function-stability tradeoffs. In contrast, new function residues as destabilizing as the “average” protein mutation, although they appear to be more destabilizing than mutations that occur during non-adaptive evolutionary changes.

### Protein Stability and the Evolution of New Functions

Although our findings indicate no specific tradeoffs between new function and stability, at the end of the day, the majority of new-function mutations are destabilizing. Furthermore, the fact that there are fewer highly destabilizing mutations amongst “successful” *new-function* mutations (Figure 3) is another manifestation of the notion that stability severely constrains adaptive evolution (i.e., the acquisition of new functions) [32]–[34]. Thus, although in principle new function mutations can be highly destabilizing, similar to mutations in key catalytic residues, such detrimental mutations are not commonly seen in proteins evolving new functions, either in nature, or in the laboratory. It also follows, that increasing the initial stability of the starting point enzyme will enable the subsequent acquisition of function altering mutations that are otherwise not tolerated [1],[2],[6],[34],[58].

The destabilizing effects of new function mutations should also be considered in view of the fact that the acquisition of new functions typically depends on multiple mutations. Indeed, proteins posses a threshold of stability that can initially buffer some of the deleterious effects of destabilizing mutations. Once this threshold is exhausted, however, protein “fitness” (i.e., expression and activity levels) is rapidly lost. This is manifested in the non-additive, or negative, epistatic effects of mutations on protein fitness—despite their ΔΔ*G* effects being largely additive [46]. Thus, as the adaptive process continues, proteins must regain stability through other mutations [32]. This scenario is evident in the role of Met181Thr mutation played in the evolution of TEM-1—(a global suppressor found in clinical isolates and directed evolution experiments, stabilizing −2.67 kcal/mol) towards the hydrolysis of a third-generation antibiotic [32]. Indeed, our analysis indicates that many of the *other* mutations seen in directed evolution experiments might play an essential role in compensating for loss of stability, and are thus involved in the process despite having no direct role in altering the activity of the evolving enzyme.

Thus, despite the fact that no specific activity-stability tradeoffs are associated with the acquisition of new functions, it appears that that the pattern of stability loss and restoration does underpin the evolution of new enzyme activities as previously noted [32]. It is clear therefore, that a more profound understanding of the dynamics and mechanism of stability restoration, and the ability to reproduce them in the laboratory, might be the key to achieving more rapid and effective enzyme evolution.

## Methods

We search the ISI web of science database for all articles containing: “directed evolution” and enzyme. The search included these terms within title, abstract, and key words, for the period of 2003 till the end of September 2007. The resulting articles were further screened for all cases amenable to our analysis; the criteria being: (i) crystal structure of the evolved enzyme at ≤2.5 Å resolution; (ii) directed evolution aimed at new substrate specificity, or catalytic activity, but not higher stability and other stability related properties such as tolerance to organic solvents; (iii) a detailed description of more than few mutations related to functional changes, typically including the description of single mutants to enable a distinction between *new-function* and *other* mutations. The screen resulted in a total of 22 enzymes and 548 mutations that were further analyzed.

The thermodynamic stability changes of mutations were computed using the protein design tool FoldX (version 2.52). We followed a four-step procedure as described in detail previously [38]–[40]. First, 3D structures were taken from the Protein Data Bank (PDB accession codes are listed in Table S1) were optimized using the repair function of FoldX. Second, structures corresponding to each of the single point mutants (including self-mutated structures) were generated by the repair position scan function of FoldX. Third, the energies for these structures were calculated using the energy calculation function of FoldX. Finally, the ΔΔ*G* of mutations were obtained by comparing the energy values of the mutant structure with those of the wild type structures. The energy values obtained by FoldX were converted to realistic values based on a normalization function obtained by fitting the experimental and computed data (ΔΔ*G^experiment^* = (ΔΔ*G^FoldX^*+0.078)/1.14) [40]. The ASA value of each amino acid residue was calculated by the web server program “ASA view” (http://www.netasa.org/asaview/). The ΔΔ*G* values obtained by FoldX were classified to 25 bins, each 1.0 kcal/mol wide, from −10 kcal/mol to 15 kcal/mol (all possible mutations with ΔΔ*G*>14 kcal/mol were classified into the 14–15 kcal/mol bin, and mutations with ΔΔ*G*<−9 kcal/mol into the (−10)–(−9) bin). The number of mutations in each bin was counted to make the distribution of ΔΔ*G*.

## Supporting Information

Figure S1 The difference of C distribution with secondary structure propensity. (A) The ΔΔ*G* distribution of each secondary structure. (B) The composed ΔΔ*G* distribution according to the secondary structure propensity of new-function mutations (Figure 1b) comparing with new-function mutations and all residues.
(0.53 MB EPS)

Table S1 Summary of enzymes.
(0.04 MB XLS)

Table S2 Point mutations included in the study.
(0.13 MB XLS)
